# A simple kit to use computational notebooks for more openness, reproducibility, and productivity in research

**DOI:** 10.1371/journal.pcbi.1010356

**Published:** 2022-09-15

**Authors:** Ludmilla Figueiredo, Cédric Scherer, Juliano Sarmento Cabral

**Affiliations:** 1 Ecosystem Modeling, Center for Computational and Theoretical Biology (CCTB), University of Würzburg, Würzburg, Germany; 2 Department of Ecological Dynamics, Leibniz Institute for Zoo and Wildlife Research (IZW), Berlin, Germany; 3 Biodiversity Modelling and Environmental Change, School of Biosciences, College of Life and Environmental Sciences, University of Birmingham, Birmingham, United Kingdom; McGill University, CANADA

## Abstract

The ubiquitous use of computational work for data generation, processing, and modeling increased the importance of digital documentation in improving research quality and impact. Computational notebooks are files that contain descriptive text, as well as code and its outputs, in a single, dynamic, and visually appealing file that is easier to understand by nonspecialists. Traditionally used by data scientists when producing reports and informing decision-making, the use of this tool in research publication is not common, despite its potential to increase research impact and quality. For a single study, the content of such documentation partially overlaps with that of classical lab notebooks and that of the scientific manuscript reporting the study. Therefore, to minimize the amount of work required to manage all the files related to these contents and optimize their production, we present a starter kit to facilitate the implementation of computational notebooks in the research process, including publication. The kit contains the template of a computational notebook integrated into a research project that employs R, Python, or Julia. Using examples of ecological studies, we show how computational notebooks also foster the implementation of principles of Open Science, such as reproducibility and traceability. The kit is designed for beginners, but at the end we present practices that can be gradually implemented to develop a fully digital research workflow. Our hope is that such minimalist yet effective starter kit will encourage researchers to adopt this practice in their workflow, regardless of their computational background.

## Author summary

The Open Science movement has been gaining track in recent years by reinforcing the bigger impact that collaborative research has: the more publicly available research there is, the easier it is to trust and build upon it. A key feature of effectively “available” and reusable research is being well documented, so it can be easily understood by those who need it. However, well documenting scientific work can be a daunting task and scientists may fall prey to workloads that are too heavy and possibly inefficient, if they are not familiar with the tools available for it. At the same time, since most research is conducted with at least one computational element (e.g., data analysis or storage of data in digital databases), the time is ripe to learn methods of documenting computational work. In this guide, we provide a minimal yet versatile set up to help scientists conduct and document their research in a more understandable, shareable, and impactful way.

## Introduction

As with other disciplines, the call for improving the computational reproducibility of ecological studies has been increasing over the years [1–6]. Reproducibility here refers to the ability to repeat a study and generate the same results by analyzing the same data set with the code and software originally used [7]. Reproducibility increases trust in the study and the main feature to achieve it, good documentation, is also important when researchers need to update a project or share it with newcomers. It is not uncommon for researchers to waste valuable time trying to understand details of their own work because it was originally poorly documented. Another common occurrence is researchers having to reinvent the wheel, i.e., recollect data and rewrite code, where shared work (data and code) could have been reused, adapted, and improved upon. Moreover, in contrast to observational studies, which, despite the existence of rigorous protocols, might be bound to specific conditions of the system during observation, the computational part of the work can be made self-contained and thus completely reproducible [1,2,4,6,7]. Nonetheless, a major obstacle to computational reproducibility of studies is the availability and quality of the data and code used in the original study. Making the data available has become mandatory for publishing in many journals, and the importance of providing the code used in both empirical and theoretical studies has been increasingly recognized [1,4], as well as the number of journals that have specific code-sharing policies [3]. In this paper, we join the increasing calls of the Open Science movement for code documentation and publication and provide a simple, yet effective way to do it. We use studies in Ecology as our source of examples, but the practices and ideas explained here could be transferred to many fields where computational, scripted work is done on nonsensitive data (as opposed to, e.g., personally identifiable human subjects, secretive information on organizations, or location of endangered species of potential commercial interest).

In Ecology, computational code is used for modeling, software development, data processing (i.e., storage, transformation, and analysis), and data presentation [2]. We focus on the use of code for modeling, data processing, and presentation. For ecological software, there is a variety of guidelines for publication (e.g., [8–11]). Moreover, the very work of software development forces authors to apply good practices established in Computer Science to facilitate understanding, such as code documentation and functional programming (i.e., writing your own functions, [9]). However, the use of computationally complicated analytical and modeling methods is increasingly common for students and researchers with little to no experience or knowledge on the good practices of computational work. Our goal with the kit presented here is to facilitate the application of good practices in a manner that does not increase the researchers’ workload. We do this by creating (i) a computational notebook, a file that contains the code and the code’s outputs along with descriptive text related to the code and supplementary to the manuscript’s main text, in a single, dynamic, and visually appealing file; and (ii) the file structure necessary to organize the research workflow around the notebook that provides easy access to all files relevant to the computational work. Despite the initial time necessary to get used to a new workflow, users with a basic understanding of code documentation and familiarity with the use of the Markdown syntax in RStudio (for R and Python) or in the Pluto package (for Julia language) should have no problem adhering to it. Nonetheless, for those who are unfamiliar, we provide video tutorials explaining how these tools work and how they are used in our workflow. Once users have understood these, our workflow should actually save time in the long run, by facilitating the maintenance of documentation and the understanding of the computational work, be it by the researchers themselves, collaborators, reviewers, or the public.

In this guide, we show the user how to set up a minimally reproducible workflow for any given project involving computational work. The workflow is easy to implement and maintain and can be set up individually or collaboratively. Moreover, we show how maintaining such documents integrates the production of research outputs (text, figures, and tables) to be included in the reporting and publication of the study. Finally, we discuss complementary practices that go beyond the scope of this article but should nonetheless be implemented and more complex ones that can be adopted once novice users have gained experience.

### The starter kit

Our kit consists of 2 functions whose files are available in the Wikimedia’s Fellows Freies Wissen repository https://github.com/FellowsFreiesWissen/computational_notebooks. The repository also has a DOI assigned on Zenodo: https://doi.org/10.5281/zenodo.6977667. The set_kit functions, written for R, Python, and Julia languages, are available in the kits folder of that repository.

*For R or Python projects*: you should install the RStudio software and download the set_kit.R file from the kits/R-Python subfolder. No additional package is required.

*For Julia projects*: install Julia [12] and the Pluto package and download the set_kit.jl file from the kits/julia subfolder.

In both cases, the function file does not have to be stored in the same folder as the project. In fact, it makes more sense to have it saved somewhere else and specify the path to the file when you call it in R, Python, or Julia. The repository’s README page also contains a summary of the explanations laid here, along with 2 videos explaining how to set up your projects, how to build your workflow, and links to all resources mentioned in the “Complementary practices” and “Next step” sections. Moreover, there is an examples folder showing how computational notebooks for a data analysis (in R) and a modeling study (in Julia) could be organized. We recommend that you refer to the examples and videos in the repository as you read this guide because they make it easier to understand how to interact with the notebooks.

### How to set up your project

To set up a project, the use of the functions is slightly different, depending on whether it is an R, Python, or Julia project. It is important to clarify that we are not referring to RStudio’s “Project” feature (.Rproj files), but rather to a study that is going to be organized around the notebook and file structure proposed by this kit.

#### For R or Python projects

Start by running source(“/your/local/path/to/set_kit.R”) in your console in RStudio, so that the function is available in your R environment. When calling the function set_kit, you must provide 3 arguments: proj_path to specify where the project’s folder should be created, proj_name to specify the name of the project folder to be created inside proj_path, and lang, to choose the project’s programming language (defaults to “r”). For example, the R project in the examples folder was created by running: set_kit(proj_name = “datastudy_r”, proj_path = “examples”).

#### For Julia projects

Load the function in your workspace with include(“/your/local/path/to/set_kit.jl”). Call set_kit, with 2 arguments: proj_path to specify where the project’s folder should be created, and proj_name to specify the name of the project folder to be created inside proj_path. For example, the Julia project in the examples folder was created by running: set_kit(proj_name = “modelstudy_jl”, proj_path = “examples”).

**Fig 1:**
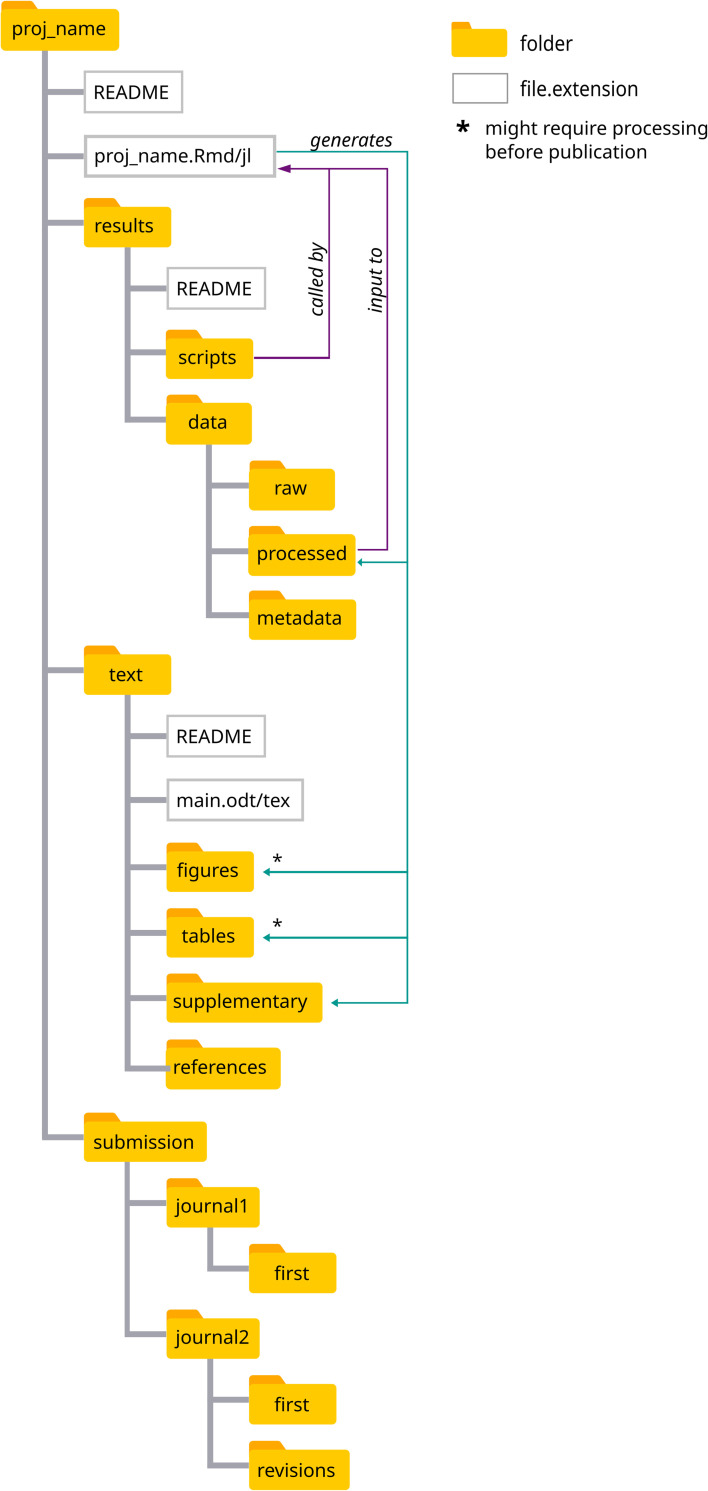
File structure created by the set_kit functions. All files relevant to a publication can be found in this nested structure. The computational notebook (proj_name.Rmd/jl) stays at the upper level, from where it has access, through relative paths, to input data that is analyzed, as well as scripts, if necessary. The outputs of such analyses can stay visible in the notebook or be saved in the figures and tables dedicated folders, also through relative paths, if they are to be included in the main text.

**Fig 2:**
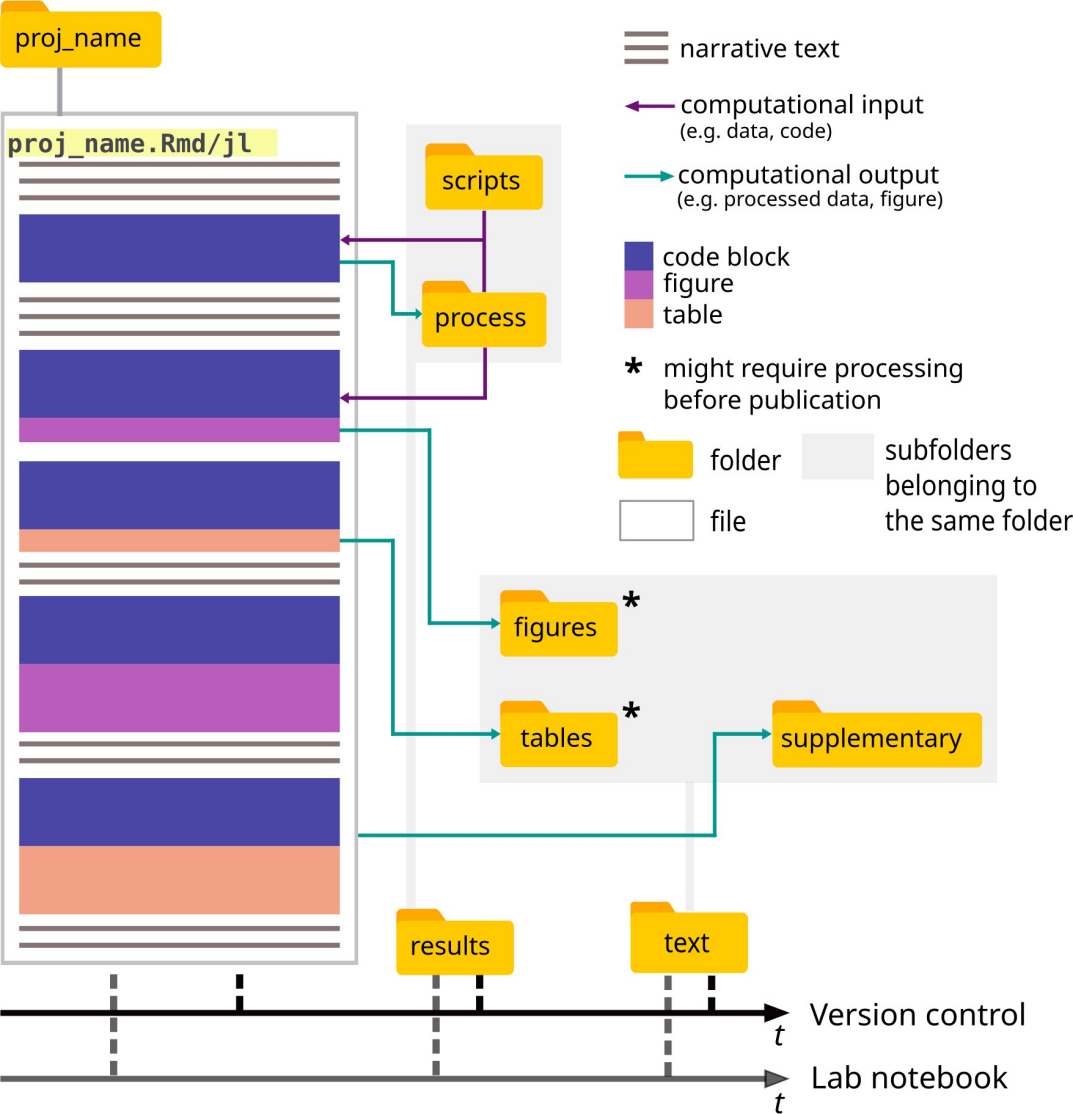
Schematics of the organization of a research project (proj_name) around a computational notebook (proj_name.Rmd/jl, referring to an RMarkdown or Julia file). The notebook contains narrative text supplementary to the manuscript’s main text, as well as text explaining the reasoning behind the computational work (included as code blocks) and its respective outputs (either kept on the notebook or saved as figures or tables). The notebook stays at upper level of a file structure (Fig 1), which provides easy access to all relevant folders for input (purple) and output (green) files through relative paths. The gray area shows subfolders that are nested inside folders (e.g., the figures and tables folders are inside the text folder). The notebook, along with all the files in the project’s folder, can be subjected to version control and be referenced in a lab notebook, thanks to the nested structure of the project. We recommend publishing it as your main supplementary material to the manuscript, along with a rendered version (.pdf or.html), any scripts, and not yet public data you use.

The following explanations are valid for both set_kit.r and set_kit.jl because the function does the same action in both programming languages:

1. Creates the folder structure depicted in Fig 1. We chose this structure (inspired by Noble [13]), because it facilitates access to all files involved in manuscript production: Files related to the main text, results, and submission are all inside their respective subfolders, inside the folder named after the proj_name argument (datastudy_r or modelstudy_jl, in the case of our examples). Therefore, this structure facilitates version control (more about it in the “Complementary practices”) and the publication of your project, since all relevant files are nested inside proj_name (Figs 1 and 2).

The results folder contains all files related to data (the primary result of research) and its organization and processing:

- data/raw: Raw data files.
- data/processed: Copies of the raw data (if that is ready to use) and the data sheets generated by any processing of the raw data.
- data/metadata: Information that will be useful for future users, readers, and reviewers of the data (e.g., description of variables names, units, and values).
- scripts: All code that is not directly written in the notebook, i.e., code that is sourced. We advise to have as much code as possible in the notebook to contribute to its narrative flow, whereby the reasoning is immediately followed by the implementation. This ensures that the reader does not have to switch to a different file to access it. However, code that is too cumbersome would contradict this advantage of notebooks and should be stored in this dedicated folder. Nonetheless, such pieces of code should be just as well documented as those included in the notebook.

The text folder contains the main text of the manuscript, with subfolders for the figures and tables included in the main text, as well as a subfolder for the supplementary material, and one for references.

The submission folder contains the files specific to journal submissions, e.g., cover letters and submitted versions.

Each of the main subfolders (i.e., proj_name, results, text, and submission) also contains a README.md file describing its contents.

2. Creates the file that constitutes the computational notebook (hereafter “notebook,” Fig 2). It is either an RMarkdown file (.Rmd) for R and Python projects or a Pluto file (.jl) for Julia. The notebook is named after proj_name (Fig 2) and remains at the top level in the project folder, with direct access to all subfolders via relative paths (e.g., results/data/processed accesses the data from the notebook). Anyone that downloads this folder can reuse the code with no need to change file paths.

Before creating the folder structure and the notebook, however, the function checks whether there already is a folder with the same name in the location you chose. If so, it sends a message asking you to change the project’s name or location. Otherwise, the function lets you know that “Your project is ready to go.”

### How notebooks work and how to build your workflow around them

**Fig 3:**
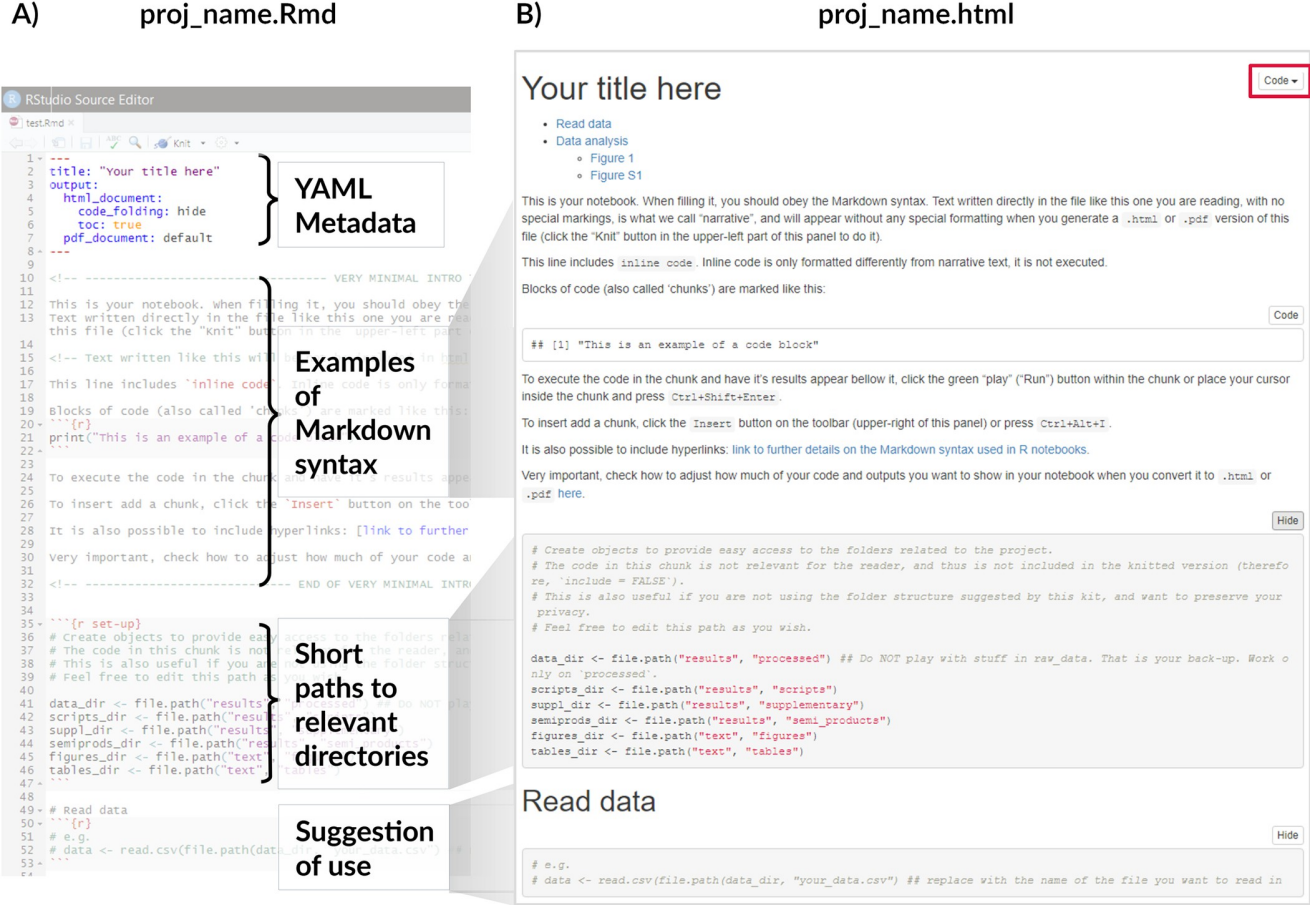
Comparison between the Rnotebook file available for editing (A) and the rendered html version (B). The function in set_kit.R creates an.Rmd file (A) with basic metadata (title and minimal formatting of the rendered file), a brief tutorial on the Markdown syntax, paths to the most relevant folders, and suggestions of use. In the.html version (B), one can see how text and code are converted and combined, as well as a couple of features available in Rnotebooks: a table of contents, just below the title (defined by the toc argument set to true in the YAML section of the Rnotebook file), and a code button, that hides the blocks of code by default, to facilitate reading (defined by the code_folding argument set to hide in the YAML section). The video accompanying this tutorial details how the document works (available in https://github.com/FellowsFreiesWissen/computational_notebooks).

**Fig 4:**
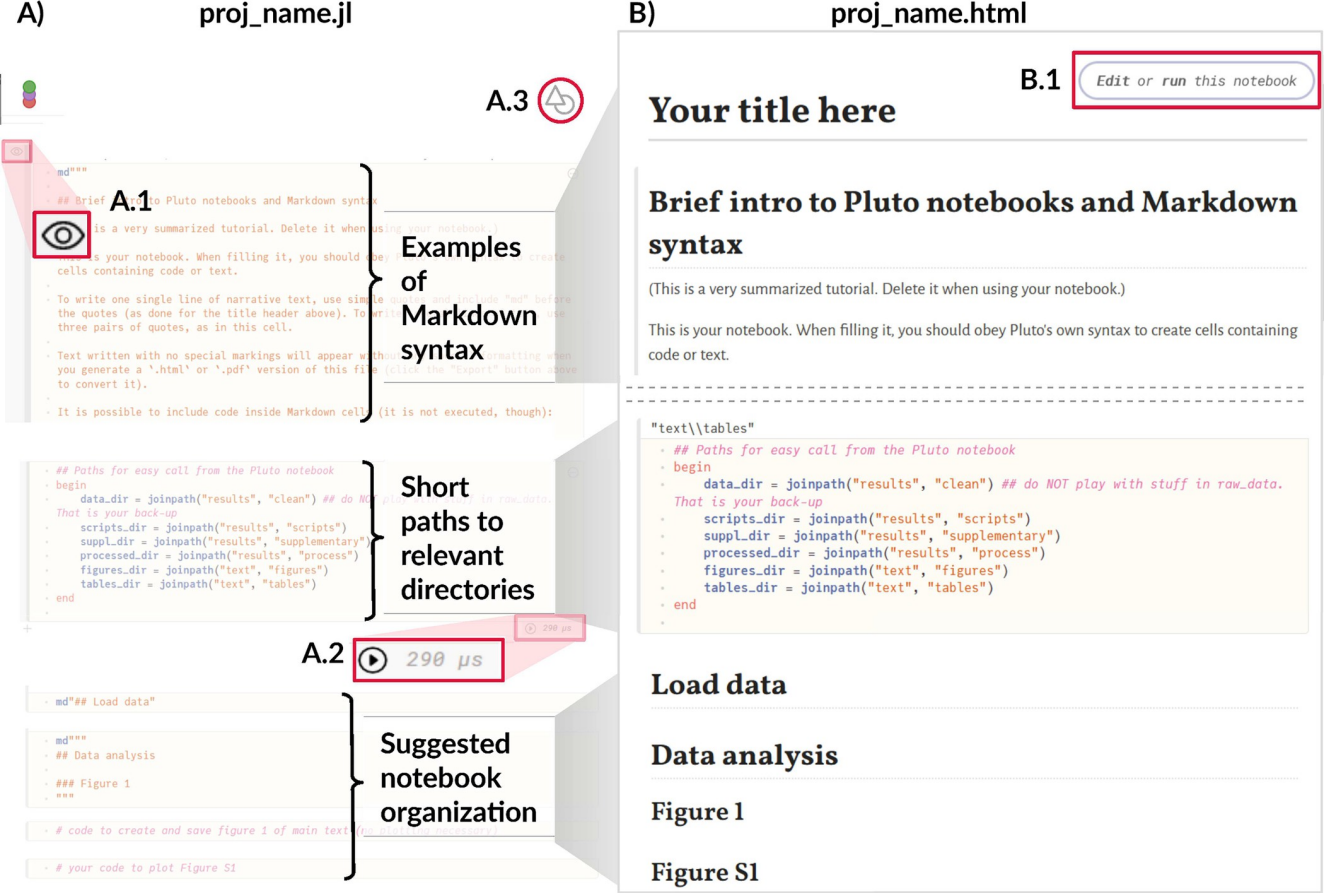
**Comparison between the Pluto notebook file available for editing (A) and the rendered html version (B).** The function in set_kit.jl creates a.jl file (A) with a brief tutorial on the Markdown syntax, paths to the most relevant folders, and suggestions of use. The Pluto notebook offers the following features: Cells of code can be hidden by clicking the “Show/hide code” button (A.1), the time for running the code is shown alongside the “Run” button (A.2), and the file type to be exported (.jl,.html, or.pdf) can be chosen by clicking the “Export” button (A.3)—no detailed formatting is possible, however. In the html version (B), a button (B.1) allows the reader to edit and run the code on Binder, a free cloud server for scientific notebooks. The video accompanying this tutorial details how the document works (available in https://github.com/FellowsFreiesWissen/computational_notebooks).

First, you should store your data into the results/data/raw subfolder. Afterwards, copy the same data into results/data/processed. No matter what you do with the copies, you should never overwrite the original files. By storing a copy of them in this dedicated separate folder and never accessing results/data/raw during the analysis, you avoid that risk entirely. Second, start filling your notebook (Figs 3 and 4).

Both notebooks (RMarkdown and Pluto) use the Markdown syntax, which supports narrative text and code in the same file, which can then be rendered as an html or pdf. A brief intro to Markdown language is available in the notebooks created by set_kit functions (Figs 3 and 4) and in the videos in the repository. In RMarkdown, any text is directly written in the file, while code to be executed is included in specially formatted areas, called *chunks* (Fig 3A). In Pluto, everything is written inside *cells*. Narrative text must be marked as md”Your text here” (use triple quotes if you want to write more than one line, Fig 4A), while executable code is written with no special marking. Both notebooks have the option of not including the code in the rendered version (Figs 3A.1 and 4A.1). Moreover, RMarkdown has a variety of controls regarding how much of the code and its outputs should be shown when rendering. In the notebook created by the function in set_kit.R, we make use of the option to include foldable code tabs in the html version (Fig 3B; this is not available for pdf).

The differences between RMarkdown and Pluto computational notebooks are mostly related to how they are displayed for editing (Figs 3A and 4A) and the options for rendering the final document (Figs 3B and 4B). RMarkdown notebooks have an *YAML* metadata section that can include information such as title, author, date, and type (html, pdf, and doc) of the rendered file, as well as formatting and aesthetics of the rendered version of the notebook (e.g., table of contents, page numbering). As of recently, YAML stands for *YAML Ain’t Markup Language*, alluding to the fact that this is a data-serialization language (designed to represent data structures) rather than a Markup language (designed to markup elements of a text). This distinction became necessary once the original meaning of the acronym, *Yet Another Markup Language*, became an erroneous definition, rather than a tongue-in-cheek reference as initially intended. For the purposes of this tutorial, understanding it as the language to control the rendering of the notebook is enough. R packages allow for further customization of the final document. For example, “bookdown” supports various formatting styles in (long-form) articles and reports (e.g., sections, headers, references, etc., [14]), and “kableExtra” allows richer formatting of tables rendered in.html and.pdf documents [15]. Pluto notebooks do not have an YAML section, are more limited in the file types (jl, pdf, and html only), and formatting (the only one currently available is shown in Fig 4B). Nonetheless, the notebook created by the set_kit.jl function is fully functional.

We suggest providing the notebook, both in original and rendered versions, as part of the supplementary material for the study being reported, since its narrative content should help guide the reader through the researcher’s reasoning (as shown in Fig 4B and in the examples folder). Readers and reviewers can then follow the work in detail, if they choose to, or just look for specific figures, tables, or bits of text. To facilitate navigation in the latter case, we suggest using descriptive names of section headers, matching the ones in the main text (e.g., place the block of code that produces a supplementary figure in a section named “Figure S1,” as shown in Fig 4B). Moreover, to avoid unnecessary repetition, figures and tables from the main text need not to be rendered in the notebook. Instead, a line of code saving them as a file is enough. In the examples folder, the notebooks were filled as supplementary material, and we detail how to work with them in the video tutorials.

We chose to use RMarkdown and Pluto notebooks for their suitability for non-experienced programmers. In case collaborators are not comfortable with these formats, a pdf version can be rendered and shared, and collaborators can add comments if necessary. Another possibility for RMarkdown is rendering, sharing, and editing a doc version of the notebook. We, however, do not encourage such practices, because the changes would have to be continuously transferred by hand between the doc/odt or pdf and the RMarkdown or Pluto files, and the authors would risk losing track of them, defeating the purpose of practicality of the kits. Moreover, it is important to remember that the notebook contains the computational work and descriptive text supporting it, not the main text of the manuscript. Therefore, the collaborators that would actively edit the notebook are the ones involved in the computational work, and thus, they are more likely to be familiar with the file format or would not have a hard time learning the basic features of the workflow we propose here.

Finally, another important feature of reproducible computational work is having information about the computational system in which the work was performed, which includes the names, versions, releases, and dependencies of the operating system and packages used when the notebook was last compiled (i.e., the code was run and the document was rendered into the published version). Both RMarkdown and Pluto contain this information. In RMarkdown, it is done by calling the Sys.info() function, which is included in a code block at the end of the document created by set_kit.R and lists the names, versions, releases, and dependencies of the operating system and packages. When you share it, readers must install the components of that environment if they want to reproduce it completely. In Pluto, the jl file has, by default, the PLUTO_PROJECT_TOML_CONTENTS section (with names and versions of packages) and the PLUTO_MANIFEST_TOML_CONTENTS section (with names and versions of indirect dependencies of the packages). Whoever opens and runs your Pluto notebook will have that environment installed by default, with no need to additionally download it.

### Complementary practices

Our kit adds to the efforts of Wilson and colleagues in promoting the “best” [9] or at least “good enough” [10] practices in scientific computing. Below, we briefly comment other practices that should be implemented alongside this kit. Since we cannot expand on how these tools work, we provide the latest comprehensive references that can support you with a low entry-level barrier.

Version control: Originally created for software development, the open source version control software *git* allows tracking and, most importantly, documenting changes to files in a directory over time. During the months or years, it takes to complete a research project, it is crucial to have a systematic, digital record of the changes in the files related to the research, as well the reasoning behind them. The directory can be stored locally on the user’s machine, or remotely, with the aid of services such as *GitHub* or *GitLab*. The nested file structure presented here can be tracked from conception until publication of the study, when the repository can be made public. As a starter tutorial oriented at scientists, we recommend Blischak and colleagues [16].

TRACE framework: For modeling work, the TRAnsparent and Comprehensive model Evaluation framework [17,18] provides a structure and a workflow to document the decisions involved in model development. In this framework, the steps involved in the “modeling cycle” (i.e., conceptual model evaluation, implementation and verification, model analysis and application, and model output corroboration) are clearly mapped to sections of a document to be written by the researcher. With such document, model users, developers, and stakeholders have comprehensive description of the model, which increases trust in the model, efficiency in analysis and development, and reuse. Ayllon and colleagues [19] provide a practical guide on how to implement this practice. We suggest using a computational notebook as your TRACE file.

### Next steps

After the simple start presented here, which should get users comfortable with the functioning of a basic reproducible workflow and the tools involved in it, users have the possibility of implementing more complex practices and tools that are beyond the scope of our paper. For example, it is possible to write manuscripts in an RMarkdown file [20], and there is a variety of online tutorials on how to format RMarkdown reports (e.g., [21–23]), as well as packages providing tools for writing reproducible manuscripts in R (e.g., [24,25]). We nonetheless still suggest using a separate computational notebook for the analytical/modeling work in these cases. Another possibility is organizing research projects as R packages, as suggested by Boettiger [26], Marwick and colleagues [27], and Hanß and Baldauf [28]. For a different workflow, the drake package [29] allows establishing a completely reproducible data analysis workflow.

Besides RMarkdown and Pluto files, 2 other publishing software are worth mentioning. The first, Jupyter notebooks [30], are long established and support a bigger range of programming languages than RMarkdown and Pluto. We do not include them in our starter kit because the user interface and, especially, version control is not as straightforward as that of RMarkdown and Pluto files. The main issue with tracking changes in Jupyter notebooks is that they cannot be easily visually inspected using *git* only. There are tools available such as *jupyterlab-git* [31] and *nbdime* [32], which provide some, but not all, functionalities of *git* in a visually appealing way. Nonetheless, they might be enough to complete the version control of a research project, so have a look into Rathi [33] for a list of tools with different capabilities. The second is Quarto [34], RStudio’s latest writing and publishing tool, which is still in its infancy. Quarto can support R, Python, and Julia code and can be edited in a variety of text editors, not only RStudio. It is a relatively new tool, which is why we did not include it in our kit. Nonetheless, it works very similarly to RMarkdown files. Therefore, users who want or need to use Quarto should be able to transfer the practices suggested here without much friction.

## Conclusion

Given the necessity of open and clear discussion of scientific ideas and the technological developments that allow the implementation of reproducible computational research workflow, it is of upmost importance that practices assuring reproducibility become common place. We hope this starter kit facilitates this change.
